# Therapeutic Potential of Volatile Terpenes and Terpenoids from Forests for Inflammatory Diseases

**DOI:** 10.3390/ijms21062187

**Published:** 2020-03-22

**Authors:** Taejoon Kim, Bokyeong Song, Kyoung Sang Cho, Im-Soon Lee

**Affiliations:** Department of Biological Sciences, Research Center for Coupled Human and Natural Systems for Ecowelfare, Konkuk University, Seoul 05029, Korea; jusink@konkuk.ac.kr (T.K.); bokysong@konkuk.ac.kr (B.S.)

**Keywords:** BVOC, terpene, terpenoid, forest aerosol, anti-inflammation

## Abstract

Forest trees are a major source of biogenic volatile organic compounds (BVOCs). Terpenes and terpenoids are known as the main BVOCs of forest aerosols. These compounds have been shown to display a broad range of biological activities in various human disease models, thus implying that forest aerosols containing these compounds may be related to beneficial effects of forest bathing. In this review, we surveyed studies analyzing BVOCs and selected the most abundant 23 terpenes and terpenoids emitted in forested areas of the Northern Hemisphere, which were reported to display anti-inflammatory activities. We categorized anti-inflammatory processes related to the functions of these compounds into six groups and summarized their molecular mechanisms of action. Finally, among the major 23 compounds, we examined the therapeutic potentials of 12 compounds known to be effective against respiratory inflammation, atopic dermatitis, arthritis, and neuroinflammation among various inflammatory diseases. In conclusion, the updated studies support the beneficial effects of forest aerosols and propose their potential use as chemopreventive and therapeutic agents for treating various inflammatory diseases.

## 1. Introduction

### 1.1. Terpenes and Terpenoids

Trees in forests are a major source of a broad range of biogenic volatile organic compounds (BVOCs) that constitute a large and diverse class of plant secondary metabolites [1,2]. The main role of these compounds is to protect plants from herbivores and pathogenic microorganisms by displaying direct toxicity, repelling herbivores, or attracting herbivores’ enemies [3,4,5,6]. It was reported that a given plant species emits a mixture of BVOCs, which can be highly complex, containing up to 200 different compounds [7].

The majority of BVOCs emitted by plants are terpenes and terpenoids. These compounds represent one of the largest groups of plant secondary metabolites, with approximately 55,000 different structures [8]. The structure of terpenes is based on the linkage of isoprene units (C_5_H_8_) such as dimethylallyl pyrophosphate and isopentenyl pyrophosphate [9,10]. These two C_5_ building blocks generate various terpene compounds by head-to-tail condensation [11]. Depending on the number of linked isoprene units, the resulting terpenes are classified into hemi-, mono-, sesqui-, di-, sester-, tri-, sesquar-, tetra-, and polyterpenes (C_5_, C_10_, C_15_, C_20_, C_25_, C_30_, C_35_, C_40_, and longer chains of C_5_, respectively) [12]. In contrast to terpenes that are hydrocarbons, terpenes containing additional functional groups, usually oxygen-containing are called terpenoids [13]. As deduced from their various structures, terpenes and terpenoids have been reported to exhibit diverse biological activities. Among them, the beneficial effects of terpene and terpenoid compounds on human health have been attracting the attention of numerous researchers, and their roles in various human disease processes, such as inflammatory diseases, tumorigenesis, and neurodegeneration, have been studied using cell and animal models for many decades, suggesting terpenes and terpenoids as potential chemopreventive and therapeutic agents for various diseases [14,15,16]. In recent years, small subgroups of novel terpenes and terpenoids have been isolated or synthesized, providing more potentially chemotherapeutic terpene compounds for clinical trials [17].

The terpenes and terpenoids in BVOCs can be classified into four groups: isoprenes, monoterpenes, sesquiterpenes, and oxygenated hydrocarbons [18,19,20]. In forested areas, monoterpenes and isoprenes are the major BVOCs that contribute to substantial emission levels to the atmosphere [21,22,23,24]. Monoterpenes are mostly emitted by coniferous species, whereas broad-leaved trees mainly emit isoprenes [25,26,27]. In contrast to the various beneficial effects of terpene compounds in human disease models, isoprenes have been recently shown to act as signaling molecules important for stress responses and growth in plants [28]; however, little is known about their positive effects on human health. Moreover, substantial emission of isoprene increases the production of ozone and smog in the presence of nitrogen oxides, due to its reactivity with hydroxyl radicals, adversely affecting the environment [29].

Various terpenes and terpenoids, mainly monoterpenes and sesquiterpenes, can be continuously emitted from specialized storage organs in leaves, stems, and trunks of trees, while others are synthesized *de novo* after invasion of a pathogen to defend the plants [30,31,32]. Due to medicinal effects of terpenes and terpenoids, forest bathing has been suggested to exhibit positive influences on human health via showering of forest aerosols containing the compounds, in addition to its physical relaxation effect [14]. This review summarizes the anti-inflammatory effects of 23 selected BVOCs from forests: 13 monoterpenes, 7 oxygenated monoterpenes, 1 monoterpene derivative, and 2 sesquiterpenes (Table 1). We surveyed the studies analyzing BVOCs emitted in various mixed as well as pure forests in North America (the United States), Europe (Estonia, France, and Turkey), or Asia (South Korea). These forest areas are located between latitudes 33 and 58 degrees in the Northern Hemisphere, mostly in the temperate zone that is also called as the mid-latitudes. These compounds were chosen because they are not only the major terpenes and terpenoids emitted in the forests, but also because as single compounds have beneficial effects on inflammatory processes with known molecular mechanisms.

### 1.2. Inflammation

Inflammation is a protective response of the host to infections and tissue damages, mediated by the activation of various immune cells [39]. However, deregulated inflammatory responses can result in acute and chronic inflammatory diseases causing excessive or long-lasting tissue damages. Macrophages play a central role in many different immune-pathological phenomena during inflammation, including overproduction of pro-inflammatory cytokines and inflammatory mediators, such as interleukin IL-1β, IL-6, tumor necrosis factor-alpha (TNF-α), and nitric oxide (NO) synthesized by inducible NO synthase (iNOS), and prostaglandin E2 (PGE-2) synthesized by cyclooxygenase-2 (COX-2). Nuclear factor-κB (NF-κB) is a central transcription factor that regulates expression of pro-inflammatory genes during inflammation [39].

In the canonical pathway triggered by Toll-like receptors (TLRs) and proinflammatory cytokines such as TNF-α and IL-1, the activated NF-κB translocates to the nucleus and regulates the expression of the pro-inflammatory genes [40]. Additionally, the pro-inflammatory mediators activate the signal transduction pathways of the core mitogen-activated protein kinases [MAPKs: extracellular signal-regulated kinase (ERK), c-Jun N-terminal kinase (JNK) and p38], which, in turn, control various cellular processes by regulating downstream protein kinases and transcription factors [41].

Several cellular processes including oxidative stress and autophagy also play an important role in inflammation. Reactive oxygen species (ROS), produced from several sources including the mitochondria, mediates the increase in leukocyte migration and junctional permeability via various signaling mechanisms [42]. In addition, a recent study showed that ROS regulates IL-1β release by directly interfering with NF-κB signaling [43]. Autophagy, a recycling process of cytosolic materials, is also closely related to inflammation. By influencing the development, homeostasis, and survival of inflammatory cells and by affecting the generation of cytokines, autophagy plays a critical role in inflammation [44]. In addition to the above processes, other pathways can be involved in inflammation progression, such as the endoplasmic reticulum (ER) stress and microRNA regulation [45]. Since these cellular processes hold such an important role in inflammation, their regulators could also modulate inflammation.

## 2. Anti-Inflammatory Effects of Volatile Terpene and Terpenoids in Forests

Studies in recent decades have demonstrated that terpenes and terpenoids ameliorate various symptoms caused by inflammation via inhibiting various steps of inflammatory processes. In this section, we categorize the anti-inflammatory activities of 23 compounds, which are the major terpenes and terpenoids emitted in the forests, into six groups and describe studies that revealed the mechanisms of their anti-inflammatory activities at the molecular level (summarized in Table 2).

### 2.1. Regulation of Pro-Inflammatory Mediators

As shown in Table 2, the majority of anti-inflammatory functions of terpenes and terpenoids have been shown to be mediated by a decrease in the levels of pro-inflammatory mediators, such as NO, interleukins, TNF-α, and PGE2. Several in vitro studies have used these pro-inflammatory mediators as biomarkers for evaluating the anti-inflammatory activity of terpenes. For example, when the RAW 264.7 cells, murine macrophages, were stimulated by lipopolysaccharide (LPS), the levels of pro-inflammatory mediators were significantly increased [125]. Pre-treatment with several terpenes including d-limonene, terpinolene, linalool, α-terpineol, α-phellandrene, and γ-terpinene, reduced LPS-induced pro-inflammatory mediator production in in vitro cultured RAW 264.7 cells [46,50,51,53,68,71]. In cultured chondrocytes, an in vitro model of arthritis-related inflammation [126], d-limonene and myrcene decreased NO and iNOS levels [49], while bornyl acetate elevated the expression of IL-11, which inhibits IL-1β-mediated up-regulation of IL-6, IL-8, matrix metallopeptidases (MMP)-1, and MMP-13 [77].

The anti-inflammatory activity of terpenes is also evaluated using various in vivo inflammatory disease models. For example, α-phellandrene, 1,8-cineole, borneol, and terpinen-4-ol inhibited the production of pro-inflammatory mediators induced by treatment with inflammatory substances, such as LPS and carrageenan, in lung injury models [52,57,59,66,70,95]. Furthermore, 1,8-cineole reduced the levels of various cytokines, such as IL-1β, IL-4, IL-6, IL-13 and IL-17A, and TNF-α in bronchoalveolar lavage fluid of asthma models sensitized with ovalbumin (OVA) or house dust mite and short-term cigarette smoke exposure of mice [56,60,61]. The inhibitory effect of 1,8-cineole on the levels of PGE2 has been also shown in an ex vivo study using blood monocytes of patients with bronchial asthma [54]. Two other terpenes, linalool and humulene, also reduced the production of pro-inflammatory mediators in an asthma mouse model sensitized with OVA [76,78]. In addition, the anti-inflammatory effects of some terpenes have also been tested in experimental colitis models. For example, borneol, terpinen-4-ol, and β-caryophyllene reduced the levels of IL-1β, IL-6, NACHT, LRR and PYD domains-containing protein 3 (NLRP3) inflammasome, in experimental colitis models induced by 2,4,6-trinitrobenzene sulfonic acid (TNBS) or dextran sulfate sodium (DSS) [64,69,79].

Based on their anti-inflammatory properties, researchers have been testing terpenes in vitro as well as in vivo to examine whether they also inhibit neuroinflammation. β-Caryophyllene has been reported to reduce inflammatory activity of BV2 microglia cells under hypoxia or after treatment with amyloid β (Aβ) peptide [84,86], while 1,8-cineole lowered the levels of NO and proinflammatory cytokines in Aβ-treated PC12 cells [58]. On the other hand, several studies have shown the effects of terpenes on the production of pro-inflammatory mediators during neuroinflammation in in vivo models. For example, d-limonene decreased NO levels in Aβ42-expressing fly heads [48], and linalool reduced the levels of the pro-inflammatory markers p38 MAPK, NOS2, COX2 and IL-1β in the brain of a triple transgenic mouse model of Alzheimer’s disease (3xTg-AD) [73]. Moreover, borneol attenuated the elevation of NO, the increase in iNOS enzymatic activity and the upregulation of iNOS expression in an ischemic model of oxygen-glucose deprivation followed by reperfusion [65].

### 2.2. Regulation of Transcription Factors Involved in Inflammatory Responses

As NF-κB is the main transcriptional factor that regulates the expression of pro-inflammatory mediators, it is frequently considered a target of anti-inflammatory molecules [127]. Consistently, a number of studies have shown that the inhibitory effects of several terpenes on the production pro-inflammatory mediators is mediated by the down-regulation of NF-κB expression or activity (Table 2). However, most studies have only shown that the translocation of NF-κB into the nucleus, the phosphorylation of I-κB and NF-κB, and the NF-κB expression levels were reduced by terpene treatment. These studies did not show that terpene-induced reduction of NF-κB activity was a direct cause of inhibition of expression of pro-inflammatory mediators. Furthermore, there was little mention of how terpenes inhibit the activity of NF-κB.

In addition, nuclear factor erythroid 2-related factor 2 (Nrf2), a transcription factor that is involved in cellular responses to oxidative damage and inflammation, is also regulated by several terpenes. Previous studies have shown that α-pinene, β-caryophyllene, borneol, 1,8-cineole, and linalool up-regulated Nrf2 activity to protect cells from oxidative damage [72,128,129,130,131]. However, in the case of terpenes other than linalool, it is unclear whether the increased Nrf2 activity by these terpenes is associated with their anti-inflammatory action. In LPS-stimulated BV2 microglia cells, linalool-induced nuclear translocation of Nrf2, and the anti-inflammatory effect of linalool was attenuated by transfection with Nrf2 siRNA [72].

### 2.3. Signal Transduction and Direct Targets of Terpene Compounds

Inflammatory responses are regulated by a variety of signaling pathways; however, surprisingly, few studies have examined the effects of terpenes on signal transduction. Mitogen-activated protein kinase MAPK signal transduction pathways, such as ERK, JNK, and p38, play an important role in inflammatory responses, and a variety of terpenes have been shown to inhibit these signaling pathways and to exert anti-inflammatory activities in various experimental models [49,66,71,87,88,89,97,100]. In addition, a recent study has reported that borneol induced the activation of M2 macrophages in a STAT3-dependent manner in a DSS-induced colitis model [96]. Unfortunately, little is known about how terpenes affect these signaling systems.

Interestingly, several terpenes, including borneol, camphor, 1,8-cineole, limonene, and linalool activate transient receptor potential vanilloids (TRPVs), a family of transient receptor potential cation channels [95,132,133,134,135]. As TRPVs have been strongly implicated in inflammatory responses, these channels are likely to mediate the anti-inflammatory action of some terpenes [136]. However, there are conflicting data on the role of TRPV channels in the inflammatory response [136], and to date, there is not much experimental evidence on their role in the anti-inflammatory action of terpenes. Meanwhile, β-caryophyllene is known as a functional agonist of cannabinoid type 2 receptor (CB(2)R) [99]. CB(2)R is the peripheral receptor for cannabinoids, which is mainly expressed in immune tissues and has been shown to modulate immune cell functions [137]. In a DSS-induced colitis model and a cisplatin-induced nephropathy model, CB(2)R mediated the anti-inflammatory activities of β-caryophyllene [100,101]. Similarly, the inhibitory effects of β-caryophyllene on the activation of NF-κB and the secretion of inflammatory cytokines were abolished by CB(2)R RNA interference in cultured microglia under hypoxia, suggesting that CB(2)R mediates anti-inflammatory responses induced by β-caryophyllene [84].

### 2.4. Function of Terpene Compounds against Oxidative Stress

Oxidative stress results from an imbalance between the production of ROS and their elimination by protective mechanisms. The excessive production of ROS causes tissue injury that may lead to the inflammatory process [42]. ROS generation occurs in two ways: it can be naturally produced within the cells and plays roles in the regulation of cell homeostasis and functions [138] or it can be produced during cell respiration in mitochondrial oxidative metabolism [139]. The main targets of oxidative stress are proteins, lipids, and DNA/RNA; modifications in these molecules may increase the chance of mutagenesis, as well as irreversible damage to cells, resulting in cell death by necrotic and apoptotic processes [140]. Terpene compounds may reduce the catalytic activity of enzymes involved in ROS generation, or may protect against oxidative damage through several mechanisms of antioxidant activity including scavenging a wide range of ROS and capacity for metal ion chelation [141,142].

Many studies have demonstrated antioxidative stress activities of volatile terpenes and terpenoids in vitro. d-Limonene is a monoterpene with powerful antioxidative properties. Apoptosis of human lens epithelial cells has been suggested as a cause of cataract. The in vitro study by Bai et al. showed that d-limonene can effectively protect the lens epithelial cells from apoptosis caused by H_2_O_2_-induced oxidative stress by inhibiting ROS generation, caspase-3/caspase-9 activation, p38 MAPK phosphorylation, and by increasing the Bcl-2/Bax ratio [103], thereby suggesting its beneficial use for treating cataracts. Furthermore, d-limonene not only induced proliferation of normal lymphocytes by decreasing H_2_O_2_ levels via increased antioxidant enzymatic activities, but also protected the cells from the oxidative stress induced by exogenous H_2_O_2_ [104], indicating the pharmacological potential of limonene on normal lymphocytes in various diseases related to oxidative stress. Similarly, myrcene, a well-known natural anti-photoaging product, decreased the production of ROS, MMP-1, MMP-3, and IL-6, and increased transforming growth factor type 1 (TGF 1) and type I procollagen secretion in UVB-irradiated human dermal fibroblasts [107]. Furthermore, myrcene treatment dramatically reduced the phosphorylation of various MAPK-related signaling molecules, suggesting its potential application in the skincare industry owing to its protective effect on photoaging. Terpinolene and α-phellandrene have also been shown to attenuate inflammation and oxidative stress in vitro by significantly inhibiting NO and superoxide production in a macrophage cell-culture-based assay [53]. In another in vitro experiment to assess antioxidant capacities, four monoterpenes, linalool, linalyl acetate, α-terpinene, and γ-terpinene, were found to scavenge peroxyl radicals, strong oxidant nitrogen radicals, weak oxidant nitrogen radicals, and lipidic peroxyl radicals, as well as to chelate oxidant metal ions [108].

In addition to their in vitro functions, antioxidative stress activities of volatile terpenes and terpenoids have also been demonstrated in vivo. Camphene, another strong antioxidant, has been assessed for its antioxidant potential using tert-butyl hydroperoxide (t-BHP)-stressed rat alveolar macrophages [106]. Camphene increased the cell viability and glutathione (GSH) content and restored the mitochondrial membrane potential, but inhibited NO release and ROS generation. Compared to other terpenes, camphene has also been found to have high scavenging activities against different free radicals generated in vitro [104,105], suggesting its possible therapeutic role in various inflammatory diseases.

Antioxidant effects of volatile terpenes and terpenoids have also been demonstrated in brain cells in vitro as well as in vivo. α-Pinene and 1,8-cineole have shown their potential in vitro antioxidant activities against H_2_O_2_-induced oxidative stress in the astrocytic U373-MG cell line by inhibiting ROS production and lipid peroxidation as well as by increasing the endogenous antioxidant status [102], suggesting the two compounds as regulators of the cellular redox balance in astrocytes. Similarly, both sesquiterpenes, humulene and β-caryophyllene, have been reported to have protective activity on cultured primary brain astrocytes against H_2_O_2_-induced cell death, possibly due to their accumulation in the cytosol [112]. β-Caryophyllene alone has shown to reduce the rate of ROS production and the associated respiratory activity albeit in an arthritic rat model [113]. In addition to those effects in astrocytic cells, another monoterpene linalool reduced cell death of immortalized neuronal HT-22, which is mediated by glutamate, an inducer of oxidative stress in neurons [111]. Furthermore, it was shown that linalool attenuates oxidative stress and mitochondrial dysfunction mediated by glutamate and N-methyl-D-aspartic acid (NMDA) toxicity, proposing its role as a neuroprotective agent against neurodegenerative brain diseases where oxidative stress contributes to their pathology. In the case of p-cymene, its antioxidant potential has been evaluated in the hippocampus of mice that were intraperitoneally treated with 0.05% Tween 80 [109]. p-Cymene significantly decreased lipid peroxidation and nitrite content, whereas significantly increased SOD and catalase activity, proposing its use as an in vivo antioxidant compound as well as a neuroprotective agent in the brain.

In contrast to their protective effects on oxidative stress, treatment of some terpenes may cause oxidative stress. Camphor is a monoterpene widely used in cosmetics, pharmaceutics, and the food industry. Agus et al. have demonstrated that it induces oxidative stress-mediated apoptotic cell death in a unicellular eukaryotic model, the fission yeast *Schizosaccharomyces pombe* [110]. In that study, camphor-induced excessive ROS production caused a dramatic increase in mortality rates due to the induction of intrinsic apoptosis revealed by mitochondrial impairment and apoptotic nuclear morphology, alerting the potential effects of camphor on apoptotic cell death. On the other hand, α-phellandrene that is widely used in the food and perfume industry has been reported to induce cell morphological changes and apoptosis in vitro in murine leukemia WEHI-3 cells [143]. Treatment with α-phellandrene-induced ROS production and cytochrome c release from mitochondria, subsequently triggering apoptosis of the tumor cells, thus suggesting its potential as an anti-tumor agent. However, interestingly, an opposite activity of α-phellandrene on wound healing has recently been reported, as it was shown to attenuate inflammation and oxidative stress in vitro [53].

### 2.5. Autophagy

Through autophagy, cells can eliminate damaged or harmful components, thus, allowing the cells to survive when responding to multiple stressors [144]. There is increasing evidence suggesting that autophagy plays a critical role in the development and pathogenesis of inflammation and immunity response [145,146].

Limonene has been shown to strongly stimulate autophagy and to prevent tumor growth in vivo as well as in vitro [114]. The treatment of limonene accelerates LC3 lipidation, which is accomplished by targeting autophagosome formation and induction of basal autophagy via activating ERK and by not inhibiting the mTOR kinase [115]. Russo et al. demonstrated that limonene increases the levels of LC3-II, a lipidated form of LC3, and subsequently stimulates autophagy to induce cell death and decrease the viability of neuroblastoma cells [116]. Another monoterpene, camphor have also been studied about its effects on autophagy in yeast *Schizosaccharomyces pombe* [119]. In this study, low-dose camphor exposure activated autophagy, confirmed by increased autophagic vesicles and transcriptional upregulation of autophagy-related gene 6 (Atg6), whereas high-dose camphor exposure resulted in dramatic cell death rates. Among yeast apoptosis mediators, allograft inflammatory factor 1 (Aif1) was found to mediate camphor-induced cell death, indicating differential regulation of autophagy and apoptosis depending on the camphor dose.

Several interesting cases of terpene usage for the development of anti-tumor drug candidates related to autophagy activities have been reported. Borneol promotes autophagy by enhancing the permeability of chemicals, especially to brain [147]. In one study, Yu et al. have investigated the effect of the treatment with the combination of tetramethylpyrazine phosphate (TMPP) and borneol on the alleviation of ischemia/reperfusion (I/R) injury [148]. The combination therapy synergistically enhanced autophagy via increasing the levels of LC3-II/I, phosphorylated pAMPK and ULK1 to protect the hypothalamus and striatum against I/R-induced apoptosis. In another study, Yu et al. have examined the effect of TMPP and borneol on the I/R-induced damage in the cortex and hippocampus [120]. This combination therapy increased the levels of pAMPK and ULK1, while it decreased mTOR to switch from apoptosis to protective autophagy. On the other hand, p-cymene can be utilized to synthesize novel and efficient metal anti-tumor drugs, ruthenium complexes. These complexes displayed lack of cytotoxicity in vivo as well as in vitro, and successfully exhibited anti-proliferative capacity associated with a combined mechanism of apoptosis and autophagy [117,118].

### 2.6. Functions of Terpenes on Other Pathways

Recently, many novel action mechanisms of volatile terpene compounds have been reported on their anti-inflammatory functions. For example, limonene treatment reduced methylglyoxal-triggered ER stress that induces inflammatory signaling in the murine preosteoblast cell line, in addition to its autophagic and ROS releasing activities [149]. In addition, α-pinene was shown to induce cell cycle arrest and anti-tumor activity by controlling miR-221 [123], expending their functions on microRNA regulation. Moreover, 3-carene as well as α-pinene were shown to function as positive modulators for benzodiazepine (BZD)-receptors, exerting a sleep-enhancing effect upon oral administration [122,124], supporting the beneficial effects of forest bathing for patients with sleep disorders or anxiety.

Recent studies have also focused on novel mechanisms associated with the activity of volatile terpene compounds on vascular- or neuroinflammation. Peroxisome proliferator-activated receptor gamma (PPARγ) is a ligand-dependent transcription factor that inhibits the expression of inflammatory cytokines and directs the differentiation of immune cells toward anti-inflammatory phenotypes [150]. Thus, natural PPARγ agonists found in foods may be beneficial to human health by acting as anti-inflammatory molecules. In an experiment involving diet-induced dyslipidemia and vascular inflammation with Wistar rats, β-caryophyllene induced the suppression of vascular inflammation via the PPARγ receptor, a treatment superior to pioglitazone, an approved PPARγ agonist [81]. As for action against neuroinflammation, Cutillas et al. demonstrated that some terpene components from Spanish marjoram suppress both lipoxygenase and acetylcholinesterase activities, among which bornyl acetate and limonene showed the highest lipoxygenase inhibition, while 1,8-cineole was the best acetylcholinesterase inhibitor [108]. 5-Lipoxygenase is a pro-inflammatory enzyme; acetylcholinesterase inhibitors were also shown to reduce the pro-inflammatory response as well as post-surgery neurodegeneration in the cortex and hippocampus [151,152]. Thus, terpene components are suggested as topics for further research into inflammatory and Alzheimer’s diseases.

## 3. Therapeutic Potentials of Volatile Forest Terpene and Terpenoids on Inflammatory Diseases: Respiratory Inflammation, Atopic Dermatitis, Arthritis, and Neuroinflammation

Currently, many therapeutic chemicals have been developed to treat inflammation, some of which induce severe side effects. Thus, the search of novel anti-inflammatory agents with fewer side effects is highly appealing. Terpene products in various forms have been used as ethnomedicines for a long time due to their beneficial healthcare effects. Individual treatment with terpene compounds as well as a number of essential oils containing monoterpenes from plants have been reported to exert anti-inflammatory activities by inhibiting various proinflammatory steps, as described above, suggesting their application potential as therapeutic agents against many inflammatory diseases. In this section, we focus on therapeutic potentials of volatile terpenes and terpenoids emitted in forested areas, which have been reported so far to be associated with four inflammatory diseases; respiratory inflammation, atopic dermatitis, asthma, and neuroinflammation. According to literature search, 12 compounds among the major types of terpene compounds emitted in forested areas have been reported to be effective in the four diseases; six terpenes (α-pinene, β-pinene, d-limonene, myrcene, α-terpinene, and β-caryophyllene) and six terpenoids (1,8 cineole, camphor, borneol, α-terpineol, linalool, and bornyl acetate).

### 3.1. Respiratory Inflammation

Asthma is a chronic respiratory condition that results in inflammation and constriction of the bronchiole airways. Despite incomplete understanding, asthma has been known to be caused by a combination of complex interactions between environmental and genetic factors [153]. Environmental factors include exposure to air pollution and allergens [154], while many different genes have been implicated as risk factors for asthma [155]. Studies on six or more separate populations revealed that asthma is associated with various genes, such as CTLA4, GSTM1, IL4R, IL10, LTC4S, and SPINK5 [156], many of which are related to the immune system or inflammation. Although symptoms can be prevented by avoiding allergens and irritants, and by the use of inhaled corticosteroids, there is no cure for asthma [157].

Several studies have shown the beneficial effects of some volatile terpene compounds via the anti-inflammation response on airway inflammation in animal models of allergic asthma. In the mouse asthma model, inhalation of limonene significantly reduced the levels of various proinflammation-related molecules as well as airway fibrosis, indicating that limonene has the potential to reduce airway remodeling [158]. Further in vivo investigation using an asthmatic rat model, a novel series of α-terpineol derivatives improved lung function as well as enhanced airway smooth muscle relaxation activity [17]. In addition, borneol and terpineol, extracted from a traditional Chinese formulation for the treatment of asthma, inhibited histamine-induced in vitro bronchoconstriction of isolated tracheal smooth muscles in guinea pigs, thus, indicating anti-asthmatic action [159].

Positive effects of volatile terpene compounds on asthma have been demonstrated in human studies as well. α-Pinene was shown as a bronchodilator in human volunteers at low exposure levels [160], making it of value to patients with asthma. Another example is 1,8-cineole. Based on its pharmacodynamic effects proven in numerous pre-clinical studies, 1,8-cineole has been shown to have clinical efficacy and therapeutic benefits in inflammatory airway diseases, such as asthma and chronic obstructive pulmonary disease in a placebo-controlled, double-blind trial [161,162].

In contrast to asthma, pneumonia is a lung infection caused by bacteria, fungi, parasites, or viruses. Like asthma, pneumonia causes lung inflammation, but, at the end, it affects the alveoli at the end of the bronchiole airways. Pneumonia can cause the lungs to fill with mucus, making breathing difficult, but it is curable. A positive effect of bornyl acetate treatment on lung inflammation has been reported in a mouse model with acute lung injury [163]. To induce acute lung injury, BALB/c mice were injected intranasally with LPS. Pretreatment with bornyl acetate downregulated the levels of proinflammatory cytokines as well as the phosphorylation of various signaling molecules related to inflammation. The bornyl acetate treatment reduced the total number of cells, neutrophils, and macrophages, and reduced histologic alterations in the lung, suggesting bornyl acetate as a preventive agent for lung inflammatory diseases.

Another condition of airway inflammation is allergic rhinitis, which is a disorder of the upper airways caused by an inflammation of the nasal membranes. Asthma and allergic rhinitis often coexist, with allergic rhinitis being a major risk factor for the occurrence of asthma [164]. For the application of terpenes on allergic rhinitis the therapeutic usefulness of α-pinene was suggested [165]. α-pinene was administered daily to mice before intranasal OVA challenge. Pretreatment with α-pinene caused a decrease in clinical symptoms. In addition, the levels of various proinflammatory mediators such as TNF-α and MIP-2, and nasal IgE declined in OVA-sensitized mice, suggesting α-pinene as a promising anti-allergic agent for the clinical management of allergic rhinitis.

In contrast to the beneficial effects described above, adverse effects of inhaled terpene compounds on airways can also be found. Oxidation products of α-pinene and d-limonene have been shown to cause irritation both in the upper airways and pulmonary regions in mice [166]. In addition, results from a population-based cross-sectional survey of the association between VOCs in residential indoor air and lung function in Canada revealed α-pinene was one among 10 chemicals that were negatively associated with lung function out of 84 VOCs examined [167].

### 3.2. Atopic Dermatitis

Atopic dermatitis (AD) is a common chronic inflammatory skin disease. Genetic susceptibility plays an important role in causing atopic dermatitis, for example, defects in skin genes important for maintaining the barrier function [168]. Cytokines are another trigger of AD, which are involved in the negative regulation of immunopathogenesis of AD [169]. Mouse models for AD can be categorized into three groups, among which is NC mouse that spontaneously develops AD-like skin lesions. Several sublines of NC mice including NC/Nga, NC/Jic, and NC/Tnd, have been developed [170]. Upon topical application of β-pinene, atopic-like skin conditions in NC/Tnd mice were ameliorated [171,172]. In the atopic dermatitis mouse model induced by 2,4-dinitrochlorobenzene, as well as in an in vitro experiment with a human adult low-calcium high-temperature keratinocytes, Cinnamomum camphora leaves, whose major component is camphor, alleviated atopic dermatitis symptoms and allergic skin inflammatory responses, such as inhibition of chemokine production as well as Jak/STAT and ERK 1/2 phosphorylation. The inhibitory effects of BVOCs such as borneol and myrcene on AD were also demonstrated in vitro. Borneol showed strong inhibitory effects on the human transient receptor potential cation channel, member A1 (hTRPA1), possibly due to its blocking action on TRPA1, expressed in sensory neurons, which functioned as a TRPA1 antagonist with anti-inflammatory and anti-allergic effects [173,174]. Myrcene ameliorates human skin aging via upregulation of MMPs production and downregulation of MAPK-related signaling molecules such as pERK, pp38, and pJNK and AP-1 [107].

However, in addition to the beneficial effects of the above volatile terpene compounds on AD, many adverse cases have also been reported. In evaluation studies using various assays, many compounds including α-pinene, β-pinene, limonene, beta-phellandrene, and camphor, have been reported to show positive allergenicity responses causing contact dermatitis [175,176,177]. Despite their beneficial activities as antioxidants, some volatile terpene compounds can autoxidize rapidly to skin allergens on air exposure. Previous studies have shown that though limonene, linalool, caryophyllene, α-terpinene are not allergenic themselves, they readily form allergenic compounds on air-exposure; and there were patients that showed a positive reaction to the oxidized products [178,179], emphasizing the need of testing with such compounds.

### 3.3. Arthritis

Rheumatoid arthritis (RA) is a chronic inflammatory autoimmune disease that is characterized by progressive destruction of articular cartilage and bone and is difficult to treat effectively. Although genetic and environmental factors have been reported to be involved in the development of RA, the pathogenesis of RA has not yet been completely understood because of its complex and multifactorial etiology. Recently, accumulating data have shown the potential role of epigenetic mechanisms in the development of RA. Although there has been significant progress toward achieving disease remission without joint deformity with the aid of anti-rheumatic drugs and pharmacologic therapies, a considerable proportion of RA patients do not effectively respond to the current therapies and thus new drugs are urgently required [180].

One of the most common experimental models for an RA study is adjuvant-induced arthritis using rats, where a strong and generalized inflammatory response is exhibited. This animal model allows researchers to investigate the pathogenesis in the joints as well as systemically, such as modifications in liver metabolism, which share features of RA seen in patients. Among the 23 volatile terpene compounds investigated in this study, β-caryophyllene has been reported as a good candidate to treat RA. Vijayalaxmi et al. evaluated the ameliorative effect of oral administration of β-caryophyllene in rats with experimental arthritis induced by Complete Freund’s adjuvant, and found that the compound significantly decreased arthritis and improved arthritis index, paw volume as well as histopathology and biochemical parameters such as anti-oxidants and serum nitrates [181]. More recently, β-caryophyllene has been found to reduce systemic inflammation in rats with adjuvant-induced arthritis and to, additionally, reduce oxidative stress in the liver and plasma of these animals without hepatotoxicity [113], suggesting β-caryophyllene as a starting point for the development of novel anti-inflammatory drugs.

In contrast to RA, osteoarthritis (OA) is a degenerative disorder including mild inflammation. Rufino et al. demonstrated the anti-inflammatory and chondroprotective effect of α-pinene in human chondrocyte, in which α-pinene prevented against IL-1β-stimulated inflammatory and catabolic pathways, such as NF-κB and JNK activation and the expression of the inflammatory (iNOS) and catabolic (MMP-1 and -13) genes [182] this suggests potential value as an anti-osteoarthritic drug. In addition, Bornyl acetate is the main volatile constituent in some Chinese traditional herbs, and Yang et al. reported its therapeutic potential in patients with OA [77]. In the study, they found that bornyl acetate elevates the expression of IL-11 in chondrocytes, and functions antagonistically on IL-1β-mediated up-regulation of IL-6, IL-8, MMP-1, and MMP-13, which play a pivotal role in OA cartilage destruction.

### 3.4. Neuroinflammation

Neuroinflammation is inflammation in the nervous tissue and is mainly mediated by microglia, the resident mononuclear phagocytes of the brain [183]. Activation by various pro-inflammatory cues, including invading pathogens, trauma, infection, and stroke, triggers neuroinflammatory responses including activation of microglia, local invasion of circulating immune cells, and production of inflammatory factors [184]. Expression of receptors for sensing potential threats and microglial pro-inflammatory cytokines, as well as production of ROS and NO, are increased in the activated microglia [185]. Although an acute inflammatory response is beneficial to neurons, chronic inflammation damages the brain; therefore, abnormal activation of neuroinflammatory processes has been implicated in neurodegenerative diseases and brain injuries [183].

Coincident with their anti-inflammatory activity in the peripheral macrophages, some terpenes have been shown to inhibit the activation of microglia under several pro-inflammatory cues in in vitro studies (Table 2). For example, β-caryophyllene inhibited release of pro-inflammatory cytokines and production of NO and PGE2 as well as the activation of NF-κB in the cultured microglia cells under hypoxia or Aβ-treatment [84,86]. Anti-neuroinflammatory activity of linalool, β-caryophyllene and borneol was also observed in the LPS-stimulated microglia cells [72,80,97]. On the other hand, eucalyptol and borneol have shown to exert their inhibitory effects on the pro-inflammatory responses in Aβ-treated PC12 cells and rat primary cultured cortical neurons with oxygen-glucose deprivation, respectively [58,65]. Based on the fact that β-caryophyllene is a ligand of CB2R that is closely related with neuroinflammation [186], most in vivo studies focused on the effect of β-caryophyllene on neuroinflammation. β-Caryophyllene inhibited neuroinflammation and showed beneficial effects in models of insulin resistance and obesity, Parkinson’s disease, and multiple sclerosis [82,83,187]. Additionally, borneol attenuated brain neuronal and microglial inflammation in LPS-induced sepsis mice [97], and d-limonene reduced glial cell number and NO levels in the brain of *Drosophila* model of Alzheimer’s disease [48].

Despite their anti-inflammatory activity in the peripheral macrophages, only a limited number of studies have shown the suppressive function of some volatile terpenes and terpenoids against neuroinflammation. However, given that many studies showed the neuroprotective properties of these compounds [14], more terpenes are expected to have neuroinflammatory activity. Therefore, further research is required in the future.

## 4. Conclusions

There has been an increase in the search of terpenes with anti-inflammatory activity in recent years. As summarized in this review, the mechanisms involved in the anti-inflammatory effects of the terpenes contained in BVOCs emitted in forests cover a wide range of targets such as transcription factors and inflammatory mediators. Although the mechanism of action of many terpenes remains to be studied, the molecular targets of terpenes are highly desirable for finding target-specific anti-inflammatory drugs. The combination of terpenes with high anti-inflammatory activity and with studied mechanisms of action, with currently used drugs could be another strategy to combat inflammatory diseases.

As more terpenoid-based clinical drugs will become available, they will play a more significant role in human disease treatment in the near future. However, some adverse effects of terpenes have been reported, depending on the concentrations of the compounds tested. For example, among the compounds described in this review, cytotoxic effects have been demonstrated for α-pinene and camphor [63,188,189]. According to the Food and Drug Administration, camphor-containing products cannot exceed 11% camphor. In addition, allergic contact dermatitis has been reported following the use of α-pinene, β-pinene, and myrcene [175,177,178,190]. This issue draws attention on the need to further explore the safe concentrations of terpenes for therapeutic proposes [191]. In this context, contact of BVOCs during forest bathing may be safer, albeit less beneficial compared to direct intake or application on skin. Taken together, this review highlights the use of the beneficial terpenes and terpenoids from forests for the management of various inflammatory-related diseases.

## Figures and Tables

**Table 1:** General types of terpenes and terpenoids emitted from forested areas. Some of the well-known synonyms and molecular formulas are shown in brackets. All structures of the compounds are from the PubChem 3D viewer database (https://pubchem.ncbi.nlm.nih.gov), in which carbons and oxygens are colored in gray and red, respectively. For more details on the drawing, refer to the description in the Pubchem web site (https://pubchem.ncbi.nlm.nih.gov/pc3d/PC3DView1.html).

Type	Name (Synonym, Molecular Formula)				Ref
Monoterpene	α-Pinene (C_10_H_16_)	β-Pinene (C_10_H_16_)	3-Carene (C_10_H_16_)	d-Limonene (C_10_H_16_)	[24,33,34,35,36,37]
	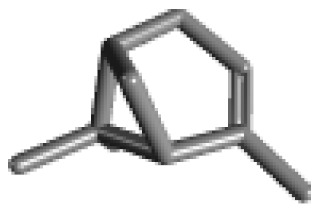	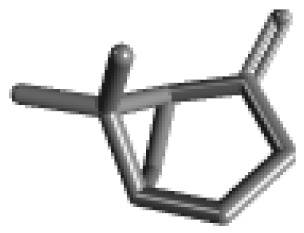	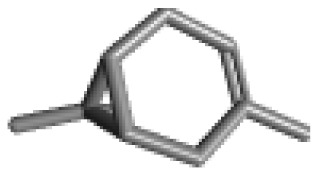	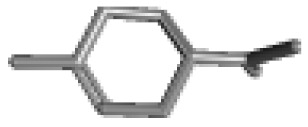	
	Camphene (C_10_H_16_)	Myrcene (C_10_H_16_)	α-Terpinene (C_10_H_16_)	γ-Terpinene (C_10_H_16_)	
	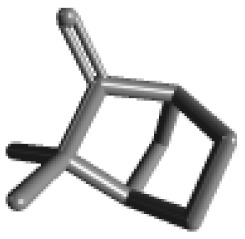	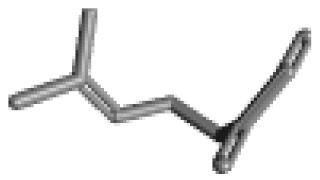	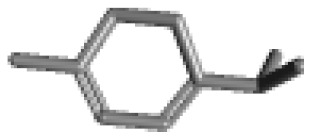	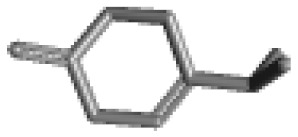	
	α-Phellandrene (C_10_H_16_)	β-Phellandrene (C_10_H_16_)	p-Cymene (C_10_H_14_)	Sabinene (C_10_H_16_)	
	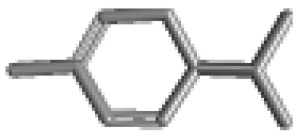	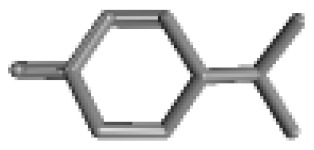	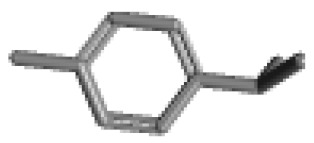	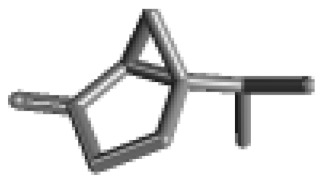	
	Terpinolene (C_10_H_16_) 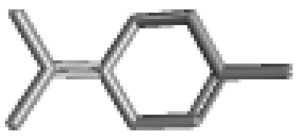				
Oxygenated monoterpene	1,8-Cineole (Eucalyptol, C_10_H_18_O)	Camphor (C_10_H_16_O)	Borneol (C_10_H_18_O)	α-Terpineol (C_10_H_18_O)	[34,37]
	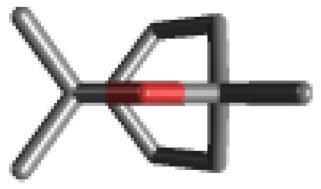	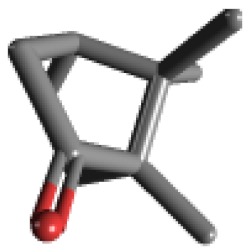	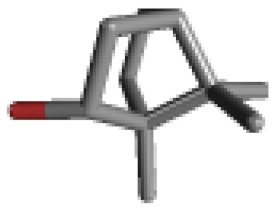	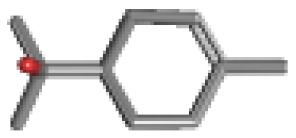	
	Terpinen-4-ol (C_10_H_18_O)	Linalool (C_10_H_18_O)	Linalool-oxide (C_10_H_18_O_2_)		
	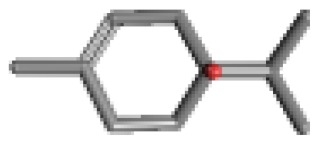	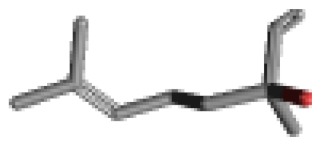	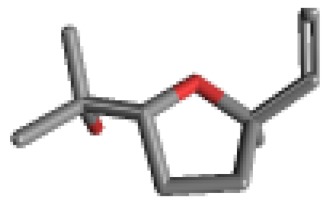		
Monoterpene derivatives	Bornyl acetate (C_12_H_20_O_2_)				[34]
	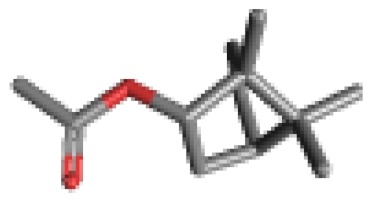				
Sesquiterpene	Humulene (α-Caryophyllene, C_15_H_24_)	β-Caryophyllene (trans-Caryophyllene, C_15_H_24_)			[34,37,38]
	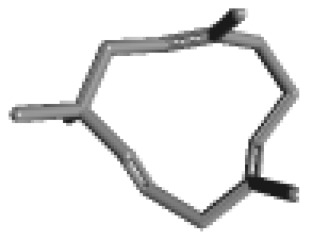	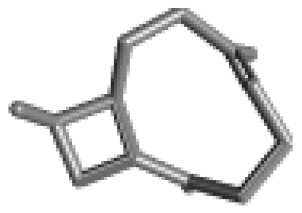			

**Table 2:** The anti-inflammatory activities of the major 23 terpenes and terpenoids emitted in the forests and their action mechanisms.

Related Inflammatory Activities	Name	Mechanism of Action	Experimental Protocol	Animal Tested	Ref.
**Pro-inflammatory mediator**	**d-Limonene**	TNF-α, IL-1β and IL-6 ↓	LPS-stimulation	Raw 264.7 cell line	[46]
		NF-kB, COX-2, iNOS and Nitrite levels ↓	Doxorubicin-induced inflammation	Wistar rats	[47]
		NO levels ↓	Aβ42 expressed heads	Fruit fly	[48]
		NO and iNOS levels ↓	In vitro treatment	Human chondrocytes	[49]
	**Myrcene**	NO and iNOS levels ↓	In vitro treatment	Human chondrocytes	[49]
	**γ-Terpinene**	TNF-α and IL-1β ↓	Carrageenan-induced peritonitis model	Swiss mice	[50]
		IL-1β, IL-6↓ and IL-10, COX-2, PGE2 ↑	LPS-Stimulation	Macrophages from mice	[51]
	**α-Phellandrene**	TNF-α and IL-6 ↓	Carrageenan injection in air pouch cavities	Wistar rats or swiss mice	[52]
		IL-6 and TNF-α ↓ NO production ↓	LPS-stimulation	Raw 264.7 cell line	[53]
	**Terpinolene**	Pro-inflammatory cytokines IL-6 and TNF-α ↓ NO production ↓	LPS-stimulation	Raw 264.7 cell line	[53]
	**1,8-Cineole**	Production of LTB4 and PGE2 from monocytes ex vivo	Stimulated with the calcium ionophore A23187 measured ex vivo	Blood monocytes of patients with bronchial asthma	[54]
		TNF-α and IL-1β, leukotriene B4 and thromboxane B2 ↓	LPS-and IL1β-stimulation in vitro	Human monocytes	[55]
		Levels of TNFα and IL-1β in BALF ↓	Experimental model of airways allergic inflammation	Ovalbumin (OVA)-challenged Guinea pigs	[56]
		TNF-α and IL-1β ↓ and IL-10 ↑	Mouse LPS-induced acute lung injury model	ICR mice	[57]
		NO ↓ TNF-α, IL-1β and IL-6 ↓	Aβ (25-35) treatment	PC 12 cell line	[58]
		MMP-9 ↓ TNF-α, IL-6 and NO ↓	LPS-induced acute lung injury mouse model	BALB/C mice	[59]
		Production of interleukin IL-4, IL-13 and IL-17A in BALF after Derp challenge ↓	House dust mite (HDM)- induced murine asthma model	BEAS-2B cell line	[60]
		IL-1β, IL-6 and TNF-α in BALF ↓	Short-term cigarette smoke (CS) exposure	C57BL/6 mice	[61]
		IL-4, IL-5, IL-10, and MCP-1 in nasal lavage fluids ↓ IL-1β, IL-6, TNF-α, and IFN-γ in lung tissues ↓	Mice infected with influenza A virus	BALB/C mice	[62]
	**Camphor**	TNF-α, IL-1β and IL-6 in Kidney, testes, liver and lung ↑	An acute administration	Wistar rats	[63]
	**Borneol**	IL-1β and IL-6 mRNA expression in colon tissue ↓	TNBS-induced colitis	ICR mouse	[64]
		The elevation of NO, the increase of inducible iNOS enzymatic activity and the upregulation of iNOS expression ↓	In vitro ischemic model of oxygen-glucose deprivation followed by reperfusion	Wistar rats	[65]
		TNF-α, IL-1β, and IL-6 ↓	Mouse LPS-induced acute lung injury model	Raw 264.7 cell line BALB/c mice	[66]
		CD16 and CD206 expressions and levels of IL-1β, IL-6, TNF-a, and IL-10 proteins ↓	LPS-stimulated mouse microglia and septic mice	C57BL/6 mice	[67]
	**α-Terpineol**	Nitrite production ↓	LPS-stimulation	Peritoneal macrophage	[68]
	**Terpinen-4-ol**	NF-κB and NLRP3 inflammasome ↓	Dextran sulfate sodium-induced colitis	C57BL/6 mice	[69]
		LPS-induced phosphorylation of IκBα and NF-κB p65 ↓ The expression of PPAR-γ ↑	Mouse LPS-induced acute lung injury model	BALB/c mice	[70]
	**Linalool**	The production of LPS-induced TNF-α and IL-6 ↓	LPS-stimulation	Raw 264.7 cell line	[71]
		LPS-induced TNF-α, IL-1β, NO, and PGE2 ↓	LPS-stimulated microglia cells.	Murine BV2 cell line	[72]
		The levels of the pro-inflammatory markers p38 MAPK, NOS2, COX2 and IL-1β ↓	Triple transgenic model of Alzheimer’s disease mice	3xTg-AD mice	[73]
		Endotoxin-induced levels of peripheral nitrate/nitrite, IL-1β, IL-18, TNF-α, IFN-γ, and HMGB-1 ↓ Nitrate/nitrite, IL-1β, TNF-α, and IFN-γ in spleen and MLNs ↓	Endotoxin-injection	C57BL/6J mice	[74]
		Microgliosis and decreased COX2, IL-1β and Nrf2 markers in the cerebral cortex and hippocampus ↓	Focal ischemia	Wistar rats	[75]
		Levels of iNOS expression in the lung tissues caused by OVA exposure ↓	Experimental model of airways allergic inflammation	OVA-challenged mice	[76]
	**Bornyl acetate**	IL-1β-mediated up-regulation of IL-6, IL-8, MMP-1, and MMP-13 ↓	In vitro treatment	Human chondrocytes	[77]
	**Humulene**	IL-5, CCL11 and leukotriene B4 levels in bronchoalveolar lavage fluid ↓ IL-5 production in mediastinal lymph nodes (In vitro assay) ↓	Experimental model of airways allergic inflammation	OVA-challenged mice	[78]
	**β-Caryophyllene**	The serum level of IL-6 protein as well as the level of IL-6 mRNA in the tissue ↓	Dextran sulfate sodium-induced colitis	BALB/c mice	[79]
		Anti-inflammatory (IL-10, Arg-1, and urea) and anti-oxidant GSH parameters ↑and the inflammatory (IL-1β, TNF-α, PGE2, iNOS and NO) and ROS biomarkers ↓	LPS-stimulation	Primary microglia cell lines (C57BL/6)	[80]
		The elevated TNF-α, NF-κB, and iNOS ↓	Rats fed a high fat/fructose diet to induce insulin resistance and obesity	Wistar rats	[81]
		The iNOS in the lumbar spinal cord ↓	Experimental autoimmune encephalomyelitis, a murine model of multiple sclerosis	C57BL/6 mice	[82]
		Pro-inflammatory cytokines and inflammatory mediators such as COX-2 and iNOS ↓	Rotenone-treated rat model of Parkinson disease	Wistar rats	[83]
		Hypoxia-induced cytotoxicity as well as IL-1β, TNF-α and IL-6 ↓	Hypoxia exposure	Murine BV2 cell line	[84]
		TNF-α and IL-1β ↓	Kainic acid-induced seizure activity and oxidative stress	Mouse model	[85]
		NO and PGE2 production ↓ iNOS and COX-2 ↓ Secretion of pro-inflammatory cytokines ↓	Aβ-treated microglia	Murine BV2 cell line	[86]
**Transcription factors**	**α-Pinene**	NF-κB ↓	LPS-stimulation	Mouse peritoneal macrophages	[87]
	**d-Limonene**	NF-κB ↓	LPS-induced acute lung injury	BALB/c mice	[88]
			Doxorubicin-induced inflammation in kidneys	Wistar rats	[47]
			In vitro treatment	Human chondrocytes	[49]
	**Myrcene**	NF-κB ↓	In vitro treatment	Human chondrocytes	[49]
	**1,8-Cineole**	Nuclear translocation of NF-κB p65 ↓ Expression of NF-κB target genes ↓ Protein levels of IκBα in an IKK-independent matter ↑ LPS-associated loss of interaction between NF-κB p65 and IκBα ↑	LPS-stimulation	U373 and HeLa cell lines	[89]
		The expression of NF-κB p65 ↓	Mouse LPS-induced acute lung injury model	ICR mice	[57]
			LPS-induced acute lung injury mouse model	BALB/C mice	[59]
			Short-term cigarette smoke (CS) exposure	C57BL/6 mice	[61]
			Mice infected with influenza A virus	BALB/C mice	[62]
	**Camphor**	The expressions of renal, testicular, hepatic and pulmonary NF-kB ↑	An acute administration in male	Wistar rats	[90]
	**Borneol**	Phosphorylation of NF-κB and IκBa ↓	Mouse LPS-induced acute lung injury model	Raw 264.7 cell line BALB/c mice	[66]
	**Linalool**	Nuclear Nrf-2 protein translocation ↑	Pneumonia model infected by *Pasteurella multocida*	A549 cell line C57BL/6J mice	[91]
		LPS-induced NF-κB activation ↓ Nuclear translocation of Nrf2 ↑	LPS-stimulated microglia cells.	Murine BV2 cell line	[72]
		CS-induced NF-κB activation ↓	Cigarette smoke -induced acute lung inflammation	C57BL/6 mice	[92]
		The activation of NF-κB ↓	Endotoxin-injection	C57BL/6J mice	[74]
		The activation of NF-κB in the lung tissues caused by OVA exposure ↓	Experimental model of airways allergic inflammation	OVA-challenged mice	[76]
	**Humulene**	The NF-kB and the AP-1 activation ↓	Experimental model of airways allergic inflammation	OVA-challenged mice	[78]
	**β-Caryophyllene**	Hypoxia-induced the activation of NF-κB ↓	Cultured microglia under hypoxia	Murine BV2 cell line	[84]
		Aβ1-42-induced phosphorylation and degradation of IκBα, nuclear translocation of p65, and NF-κB transcriptional activity ↓	Aβ-treated microglia	Murine BV2 cell line	[86]
**Signal transduction**	**α-Pinene**	ERK and JNK ↓	LPS-stimulation	Mouse peritoneal macrophages	[87]
	**d-Limonene**	p38, JNK, ERK ↓	LPS-induced acute lung injury	BALB/c mice	[88]
		p38 and JNK activation ↓	In vitro treatment	Human chondrocytes	[49]
	**Myrcene**	p38 and JNK activation ↓	In vitro treatment	Human chondrocytes	[49]
	**1,8-Cineole**	Phosphorylated JNK in U373 cells ↓	LPS-stimulation	U373 and HeLa cell lines	[89]
		TREM-1, NLRP3 of the inflammasome ↓ Phosphorylation of the transcription factor NF-κB and p38↓MKP-1 phosphatase, a negative regulator of MAPKs ↓	LPS-induced the murine lung alveolar macrophage inflammation model	MH-S cell line	[93]
		NLRP3 inflammasome activation and pro-inflammatory cytokine productions induced by MSU in ankle tissues in vivo ↓ MSU-induced upregulation of TRPV1 expression in ankle tissues and dorsal root ganglion neurons innervating the ankle ↓	A mouse model of gout arthritis was established via MSU injection into the ankle joint	BALB/c mice	[94]
		Inflammatory cytokines (IL-1β, TNF-α and IL-6) ↓	LPS-induced pulmonary inflammation	C57BL/6	[95]
	**Borneol**	Phosphorylation of p38 and JNK ↓	Mouse LPS-induced acute lung injury model	Raw 264.7 cell line BALB/c mice	[66]
		The activation of M2 macrophages in a STAT3-dependent manner ↑	DSS-induced colitis	Raw 264.7 cell line	[96]
		NF-κB and p38 signaling ↓	LPS-stimulated microglia	C57BL/6 mice	[97]
		TRPA1 mediated cationic currents ↓	In vitro treatment	In heterologous expression systems like Xenopus oocytes and in neurons cultured from trigeminal ganglia	[98]
	**Linalool**	Phosphorylation of IκBα protein, p38, c-JNK, and ERK ↓	LPS-stimulation	Raw 264.7 cell line	[71]
	**β-Caryophyllene**	Functional agonist of CB(2)R	LPS-stimulation	CB2-expressiong HL60 cell line	[99]
		Activation of ERK 1/2, NF-κB, IκB-kinase α/β ↓ Involvement of CB2 and the PPARγ pathway	DSS-induced colitis	CD1 mice	[100]
		Cisplatin-induced renal inflammatory response (chemokines MCP-1 and MIP-2, cytokines TNF-α and IL-1β, adhesion molecule ICAM-1, and neutrophil and macrophage infiltration) through a CB(2)R-dependent pathway ↓	Cisplatin-induced nephropathy model	C57BL/6J	[101]
		Activation of NF-κB and the secretion of inflammatory cytokines ↓	Hypoxia exposure	Murine BV2 cell line	[84]
**Oxidative stress**	**α-Pinene**	ROS formation and lipid peroxidation induced by H_2_O_2_-stimulated oxidative damage ↓	H_2_O_2_-stimulated oxidative stress	U373-MG cells	[102]
	**d-Limonene**	ROS formation/ caspase-3/caspase-9 activation/p38 MAPK phosphorylation ↓ The Bcl-2/Bax ratio induced by H_2_O_2_-stimulated oxidative damage ↑	H_2_O_2_-stimulated oxidative stress	Human lens epithelial cells	[103]
		Catalase and peroxidase activities of cell antioxidant enzymes ↑	Lymphoid cell suspensions from lymph nodes	BALB/c mice	[104]
	**Camphene**	Strong antioxidant effect and high scavenging activities against different free radicals	The nonenzymatic antioxidant capacity	Swiss mice	[105]
		The cell viability and GSH content and restored the mitochondrial membrane potential ↑ NO release and ROS generation ↓	t-BHP stressed alveolar macrophages	Wistar rats	[106]
	**Myrcene**	ROS, MMP-1, MMP-3, and IL-6, and increased TGF 1 and type I procollagen secretions ↓ The phosphorylation of various MAPK-related signaling molecules ↓	In vitro treatment	UVB-irradiated human dermal fibroblasts	[107]
	**α-Terpinene**	The best antioxidant compounds in ABTS, chelating power and DPPH assays	In vitro antioxidation assay		[108]
	**γ-Terpinene**	The best antioxidant compounds in ABTS and DPPH assays	In vitro antioxidation assay		[108]
	**α-Phellandrene**	The intracellular oxidative stress environment ↓	Mice leukemia	WEHI-3 cell line	[108]
		O2-production ↓	LPS-stimulation	Raw 264.7 cell line	[53]
	**p-Cymene**	SOD and catalase activity significantly ↑	Intraperitoneal treatment with 0.05% Tween 80	Swiss mice	[109]
	**Terpinolene**	O2-production ↓	LPS-stimulation	Raw 264.7 cell line	[53]
	**1,8-Cineole**	ROS formation and lipid peroxidation induced by H_2_O_2_-stimulated oxidative damage ↓	H_2_O_2_-stimulated oxidative stress	U373-MG cells	[102]
	**Camphor**	Excessive ROS production and mitochondrial impairment ↑	Oxidative stress-mediated apoptotic cell death	*Schizosaccharomyces pombe*	[110]
	**Linalool**	The best antioxidant compounds in ORAC and Chelating power assay	In vitro antioxidation assay		[108]
		Oxidative stress and mitochondrial dysfunction mediated by glutamate and NMDA toxicity ↓	Oxidative stress and mitochondrial dysfunction	HT-22 cells	[111]
	**Humulene**	H_2_O_2_-induced astrocytic cell death ↓	Primary astrocytes from cerebral cortices	Neonatal wistar rats	[112]
	**β-Caryophyllene**	H_2_O_2_-induced astrocytic cell death ↓	Primary astrocytes from cerebral cortices	Neonatal wistar rats	[112]
		Rates of ROS production and the associated respiratory activity in freshly isolated hepatic mitochondria ↓	Development of adjuvant arthritis	Holtzman rats	[113]
**Autophagy**	**d-Limonene**	Expression of apoptosis and autophagy-related genes ↑	In vitro and vivo treatment	BALB/c mice A549 and H1299 cell lines	[114]
		LC3 lipidation ↑ and clonogenic capacity ↓	In vitro treatment	SH-SY5Y, HepG2 and MCF7 cell lines	[115]
		LC3 II↑ and p62 levels ↓	In vitro treatment	SH-SY5Y and MCF7 cell lines	[116]
	**p-Cymene**	Autophagolysosomes ↑ and proliferation ↓ Anti-tumor metallodrug candidates	In vitro treatment	A2780 ovarian and MCF7 and MDAMB231 breast	[117]
		Autophagy with materials containing Ru complex ↑	In vitro treatment	B16 and B16-F10 cell lines	[118]
	**Camphor**	Autophagy and apoptotic cell death ↑	In vitro treatment	*Schizosaccharomyces pombe*	[119]
	**Borneol and TMPP**	LC3 II/I, pAMPK, mTOR, and ULK1 in hypothalamus, and pAMPK, mTOR, ULK1, Beclin1, and Bax in striatum ↑	Surgical induction of GCIR	Sprague-Dawley rats	[118]
		Cortex autophagy by modulating pAMPK in the pAMPK-mammalian target of mTOR-ULK1 signaling pathway ↑			[120]
	**Borneol and Luteolin**	E1, p62, and ubiquitin levels ↓ Accumulation of toxic aggregates, cell death ↑	In vitro treatment	HepG2 cell line	[121]
**Other activities**	**α-Pinene**	Sleep enhancing property through a direct binding to GABAA BZD receptors	Pentobarbital-induced sleep	ICR and C57BL/6N mice	[122]
		G_2_/M-phase cell cycle arrest *miR-221* expression level ↓ CDKN1B/p27-CDK1 and ATM-p53-Chk2 pathways ↑	In vitro treatment	HepG2 cell line	[123]
	**3-Carene**	sleep duration ↑ and sleep latency ↓ GABAA receptor-mediated synaptic responses ↑	Pentobarbital-induced sleep	ICR and C57BL/6N mice	[124]
	**1,8-Cineole**	Acetylcholinesterase activities ↓	In vitro antioxidation assay		[108]
	**Bornyl acetate**	Lipoxygenase ↓	In vitro antioxidation assay		[108]
	**Limonene**	Lipoxygenase ↓	In vitro antioxidation assay		[108]
	**β-Caryophyllene**	VCAM-1 ↓, and restored vascular eNOS/iNOS expression balance PPAR-γ agonist	high fat/fructose diet-induced dyslipidemia and vascular inflammation	Wistar rats	[81]

↓ denotes decreased activity; ↑ denotes increased activity.

## Abbreviations

AD	atopic dermatitis
Aif1	allograft inflammatory factor 1
Aβ	amyloid β
BVOCs	biogenic volatile organic compounds
BZD	benzodiazepine
CB(2)R	cannabinoid type 2 receptor
COX-2	cyclooxygenase-2
DSS	dextran sulfate sodium
ER	endoplasmic reticulum
ERK	extracellular signal-regulated kinase
GSH	glutathione
hTRPA1	human transient receptor potential cation channel, member A1
I/R	ischemia/reperfusion
IL-1β	interleukin-1β
IL-6	interleukin-6
iNOS	inducible NO synthase
JNK	c-jun N-terminal kinase
LPS	lipopolysaccharide
MAPK	mitogen-activated protein kinase
MMP-1	matrix metallopeptidase-1
NF-κB	nuclear factor-κB
NLRP3	NACHT, LRR and PYD domains-containing protein 3
NMDA	N-methyl-D-aspartic acid
NO	nitric oxide
Nrf2	nuclear factor erythroid 2-related factor 2
OA	osteoarthritis
OVA	ovalbumin
PGE-2	prostaglandin E2
PPARγ	peroxisome proliferator activated receptor gamma
RA	rheumatoid arthritis
ROS	reactive oxygen species
t-BHP	tert-butyl hydroperoxide
TGF 1	transforming growth factor type 1
TLRs	toll-like receptors
TMPP	tetramethylpyrazine phosphate
TNBS	2,4,6-trinitrobenzene sulfonic acid
TNF-α	tumor necrosis factor-alpha
TRPVs	transient receptor potential vanilloids
